# Volatile and Sensory Characterization of La Mancha Trujillo Melons over Three Consecutive Harvests

**DOI:** 10.3390/foods10081683

**Published:** 2021-07-21

**Authors:** M. A. Ferrer Valverde, E. Sánchez-Palomo, M. Osorio Alises, C. Chaya Romero, M. A. González-Viñas

**Affiliations:** 1 Area of Food Technology, Faculty of Chemical Sciences, University of Castilla-La Mancha, Avda. Camilo Jose Cela, 10, 13071 Ciudad Real, Spain; ManuelAngel.Ferrer@alu.uclm.es (M.A.F.V.); Maria.Osorio2@alu.uclm.es (M.O.A.); MiguelAngel.Gnzalez@uclm.es (M.A.G.-V.); 2 Department of Agricultural Economics, Statistics and Business Management, Universidad Politecnica de Madrid, Escuela Técnica Superior de Ingeniería Agronómica, Alimentaria y de Biosistemas, Ciudad Universitaria s/n, 28040 Madrid, Spain; carolina.caya@upm.es

**Keywords:** Trujillo melons, aroma compounds, gas chromatography-mass spectrometry, sensory analysis

## Abstract

In this work, Trujillo melons were harvested across three years (2011–2013) in La Mancha region. Instrumental and sensory analysis were used for studying Trujillo melons. Solid phase extraction (SPE) was used for isolating free aroma compounds, and then, they were analysed by gas chromatography coupled with mass spectrometry (GC/MS). Fifty-five (55) volatile compounds were identified and quantified in La Mancha Trujillo melons over this three-year period. Experienced tasters evaluated the sensory profile of Trujillo melons, and it was characterized by jam/marmalade, cucumber, fresh fruit, sweet, green, honey and ripe fruit aroma descriptors and sweet, honey, jam/marmalade, cucumber, fresh fruit ripe fruit, spice and green flavour by mouth descriptors. This study represents the first complete aromatic characterization of Trujillo melons from La Mancha region. The obtained data suggested that these melons presented a great aromatic profile and that they represent a viable alternative for expanding the traditional market.

## 1. Introduction

Melon (*Cucumis melo* L.) is one of the most economically important and widely grown horticultural crops in the La Mancha region of Spain. The main determinants of fruit quality perceived by consumers are attributes such as textural properties, flavor and visual appearance, but mainly the sensory characteristics of aroma determined by volatile compounds [1,2,3,4,5,6]. These volatile compounds, which are mainly formed from amino acids [7], have different degrees of volatility, and mainly depend on the physiological behavior and cultivar of the fruit [2,8].

It has been shown that, in climacteric genotypes, such as Galia or cantaloupe melons, esters and sulfur-derived ester compounds are the main volatiles analyzed [9,10]. About 240 compounds have been identified, mainly esters, aldehydes, alcohols, sulfur-derived compounds and [8,11,12,13].

On the contrary, non-climacteric melons, such as piel de sapo, Rochet, honeydew or casaba type, generally present lower levels of aroma volatiles, emphasizing saturated and unsaturated C_9_ alcohols, aldehydes with absence of sesquiterpenes and esters, or at least have lower levels of detection [9,14,15,16,17] and only 42 compounds have been identified.

In Spain, the largest areas dedicated to melon crops are concentrated in the southern half and principally are found in Castilla-La Mancha region. In the province of Ciudad Real, situated in this autonomous region, melon is recognized by an official Protected Geographical Indication (PGI) and is one of the most important summer crops from an economic perspective. For this reason, the aims of this research were to characterize the volatile compounds and sensory profile of “piel de sapo” type of melon cv. Trujillo (*Cucumis melo* L.) cultivated in La Mancha region (Spain) over three consecutive harvests and to determine the relationships between volatile compounds identified and quantified by GC-MS and aroma attributes were studied by the application of partial least squares (PLS).

## 2. Materials and Methods

### 2.1. Experimental Design

Field trials were performed at La Entresierra field station at Ciudad Real in central Spain (3°56′ W; 39°0′ N; 640 m altitude), between May and September of the years 2011, 2012 and 2013. The soil was a shallow sandy loam, classified as Petrocalcic Palexeralfs, with a very low variability in the first 0.60 m. Below this soil layer, it is localized a discontinuous and fragmented petrocalcic layer, and cracks within this layer provide higher vertical permeability locally. In this area there is a Mediterranean climate, with a strong continental character and widely fluctuating daily temperatures.

Melons were cultivated during the dry season, with an annual average rainfall of 400 mm. The total reference evapotranspiration (ETo) over the crop cycle ranged from 638.2 to 690.5 mm. During the three years prior to the experiment, the plots did not receive any organic or fertilizer amendments and were used to grow non-irrigated winter wheat (*Triticum aestivum* L.).

The type of melon which was used for this investigation was “piel de sapo” cv. Trujillo (*Cucumis melo* L.). In every year of experimentation, melon seeds were germinated from April under greenhouse conditions, until they had sprouted two or three real leaves. Subsequently, the seedlings were transplanted (25 May 2011, 23 May 2012 and 27 May 2013) onto plastic mulch at a density of 4444 plants ha^−1^ (1.5 by 1.5m). The mineral fertilizer was applied in the form of ammonium nitrate, and the melon received 120 kgha^−1^ of phosphorus fertilizer (phosphoric acid) for the period, added to the irrigation water and injected daily.

It was set a regular program of disease and insect control throughout the growing period, according to standard management practices.

The harvest of melons took place weekly when there were a significant number of fully ripe fruits in the field. At the moment of the harvest each melon was weighed to determine the total fruit yield (FY). The melons that were harvested were uniform, with no defects and with a weight of about 3.5 kg. They were washed, brushed, and stored at 15 °C during 2 days until analysis were done.

### 2.2. Preparation of Melon Juice

Five melons were rinsed in cold running tap water, the skin (0.8 cm) and the seeds were removed, and the remaining fruit was chopped and blended in a food processor. Portions of 100 g were weighed into polypropylene centrifuge bottles and these bottles were centrifuged at 12,000 rpm for 20 min at 2 °C in a Medifriger BL-S centrifuge (Selecta, Barcelona, Spain). For chemical analysis, the supernatant juice was filtered using a Whatman filter No. 1 (GE Healthcare UK Ltd., Buckinghamshire, UK), in order to remove any tissue particles, and the filtrate was used for all the analyses.

### 2.3. Conventional Analysis of Juice Melon

Total acidity, °Brix and pH, were measured according to AOAC method [18].

### 2.4. Extraction of Volatile Compounds of Juice Melon

A sample of 100 mL of juice melon added of 40 µL of internal standard (4-nonanol) were isolated by adsorption/desorption on preconditioned polypropylene-divinylbenzene cartridges (LiChrolut EN, 0.5 g of solid phase, Merck, Darmstadt, Alemania). After passing the melon juice samples, in order to remove sugars and other low-molecular-weight polar compounds [19], the cartridges were cleaned with 50 mL of milli-Q water.

10 mL of dichloromethane were used to elute the free fraction. To separate the frozen water from the organic phase by decantation, all dichloromethane extracts were cooled to −20 °C and then dried over anhydrous sodium sulphate. Using nitrogen stream, the organic phase was concentrated to a final volume of 200 μL.

### 2.5. Gas Chromatography-Mass Spectrometry (GC-MS) Analysis

A model 6890 N gas chromatograph quipped with a BP-21, polyethylene glycol TPA- treated capillary column (60 mm × 0.25 mm i.d.; 0.25 µm film thickness) coupled to a model 5973 inert mass selective detector (Agilent, Las Rozas, Madrid, Spain) was used. Operating conditions were the following: oven temperature program was 70 °C (5 min.)–1 °C/min −95 °C(10 min)–2 °C/min–200 °C (40 min). Injector and transfer line temperatures were 250 °C and 280 °C, respectively. Mass detector conditions were electron impact (EI) mode at 70eV; source temperature: 178 °C; scanning rate: 1 scan/s; mass acquisition: 40–450 amu. One microliter (1 µL) was injected in splitless mode. Carrier gas was helium (1 mL/min).

Identification and confirmation of the volatile compounds identified was done by using their retention times, a Wiley mass-spectral library search and pure volatile compounds. When the authentic standard was not available, the identification was based on the comparison with the spectral data of the Wiley A library and chromatographic data from the literature. The relative content of each volatile was calculated as 4-nonanol (internal standard, 1g/L) equivalent by the total ion count (TIC) peak area.

### 2.6. Sensory Descriptive Analysis

A trained panel of 15 experienced testers (nine female and six male) ranging in ages from 26 to 45 years was used to evaluate La Mancha Trujillo melons in duplicate. Availability and motivation were taking in account to select panelists. This activity was not remunerated. Judges’ selection criteria were established according to UNE 87024-1 1995 [20].

Training was carried out during four months in a two sessions per month program based on the time availability of the assessors. Along the training sessions the assessors generated a total of 21 descriptors of aroma and flavour by mouth feel of Trujillo melons. Then, the descriptors which would be used for the sensory descriptive analysis were selected by deliberation of the panelists, taking consensual decisions. The attributes that were considered inappropriate, confusing, or redundant were rejected. Finally, eight olfactory attributes and 11 flavour by mouth attributes were considered the best ones to describe the sensory characteristics of Trujillo melons.

Before formal sessions, the melons held placed for 2 h at 23 °C to enhance the perception of aroma and taste characteristics. The flesh of five fruits from each treatment was cut into 2 cm × 2 cm pieces and placed on a glass plate 30 min before sensory analysis began The sessions took place in a standard sensory-analysis chamber [21] equipped with separated booths. Light was uniform, there was absence of noise and distracting stimuli, and ambient temperature was between 19 and 22 °C across the day.

A 10 cm unstructured scale was used by the panelists to rate the intensity of each attribute. The left-hand end of the scale was “attribute not perceptible” and the right-hand end was “attribute strongly perceptible”.

### 2.7. Statistical Analysis

The statistical treatments were performed using SPSS 23.0 for Windows statistical package (IBM SPSS Statistics, Madrid, Spain). In order to determine significant differences in the concentration of volatile compounds and in the mean intensity of aroma sensory attributes of La Mancha Trujillo melons, an ANOVA test was made. When there was a significance difference between the samples, a Student-Newman-Keuls test was conducted with the level of significance set at *p* < 0.05 to determine between which groups there were significant differences. The principal component analysis (PCA) employs a mathematical procedure that transforms a set of potentially correlated response variables into a new set of non-correlated variables [22].

In order to evaluate the relationship between aroma sensory attributes and volatile compounds, partial least squares regression (PLS) was applied using XLSTAT (Paris, France). PLS shows the relationship between X data (volatile compounds) and Y data (sensory descriptor). It reduces the X variables to a set of no correlated factors that describe the variation in the data, so it is a data reduction method.

## 3. Results

### 3.1. General Ccomposition of Melons

The results about the general composition of melons is shown in Table 1. The soluble solids contents (°Brix) of samples ranged from 11.4 °Brix for 2011 year to 15.7 °Brix for 2013 year. Generally, it was found that the levels of soluble solids for 2011 melons were much lower than in others; however, there was not found significant differences ç between 2012 and 2013 melons (Table 1).

As it was expected, total acidity of melons was lower in 2013. Weather conditions can be the reason why differences were observed in the general composition of the samples of La Mancha Trujillo melons.

### 3.2. Volatile Compounds of Melon

Dichloromethane was used to extract free volatile melon juice compounds. Table 2 presents the quantitative data for the volatile compounds found in the aroma extract of melon juice grown in La Mancha region. The data is expressed as averages (µg/L) of duplicate extractions that were analyzed using *GC-MS*. The identification and quantification of 56 volatile compounds in Trujillo juice melon was due to the improvements that have been done in the analytical method of extraction. These compounds include aldehydes, alcohols, acids, benzenic compounds, esters and terpenes. The obtained qualitative and quantitative composition of the volatile fraction of the control agreed with the results of [23].

Aldehydes with low molecular weight can be related to immature stages in cantaloupe melons [11]. However, in the studied samples, most aldehydes were present at high levels or had a remarkable increasement during early growth stages and then decrease as harvest maturity increased [11]. In this research aldehydes are present in approximately 15% of the total concentration of volatile compounds of aroma profile, highlighting (*E,Z*)-2,6-nonadienal as the aldehyde in the highest concentration. Musk melon-like or musky aroma is principally provided by this aldehyde [8].

Many flavor aldehydes that were found in La Mancha Trujillo melons, such as hexanal, (*E*)-2-hexenal, (*E*)-2-heptenal, nonanal, (*E*)-2-nonenal, (*Z*)-2-nonenal and (*E,Z*)-2,6-nonadienal were also detected in cucumbers [24].

The main identified compounds were alcohols in the samples ranging from 410.89 µg/L (2013 year) to 432.37 µg/L (2011 year). Among them, (*Z*)-3-nonen-1-ol, (*Z,Z*)-3,6-nonadien-1-ol, 1-nonanol, 3-penten-1-ol, (*E*)-6-nonen-1-ol, and 4-methyl-2-pentanol were the most abundant. Unsaturated C_9_ alcohols are considered as the most important contributors to honeydew melon aroma due to their low odor threshold [25]. (*Z,Z*)-3,6-Nonadien-1-ol is classified as a grassy boiled leaf-like aroma [8] and showed the higher concentration of this group of volatile compounds. “Green notes” in muskmelon were attributed to 1-hexanol, 2-ethylhexanol and (*Z*)-3-hexenol [26], but in this investigation, these compounds showed a low concentration.

In agreement with Perry et al. [14], in honeydew melons there were only a few esters identified in La Mancha Trujillo melons. In this study esters and acetates were quantitatively, in number and levels, very low according to the literature for inodorous melons [23]. In cantaloupe melons and other climacteric melons, acetates are the major volatiles [27], whereas in other less climacteric melons, the compounds that dominated the volatile profile were aldehydes and alcohols.

Other volatile compounds which also have low odor thresholds and were identified as key odorants in the aroma profile of melon fruit, especially cantoloupe, were sulfur-containing compounds [28], but they were not detected in the aroma compounds of La Mancha Trujillo melons.

**Figure 1:**
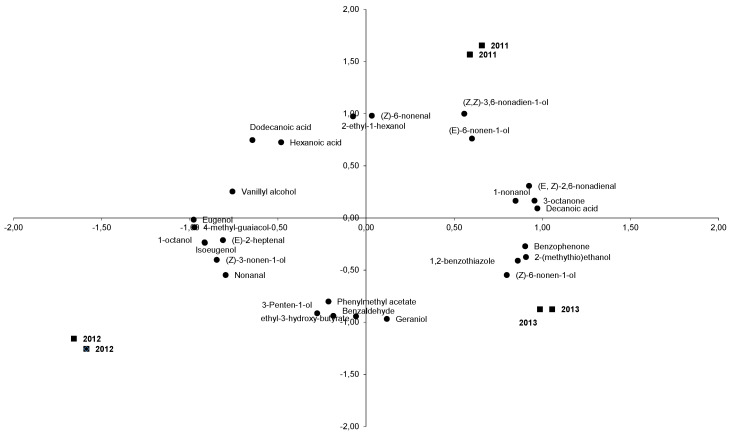
Distribution in the consensus space, the melon samples are written in bold and the vola Table 1. and axis 2 represents Dimension 2.

A principal component analysis (PCA) was applied to all volatile compounds identified and quantified in La Mancha Trujillo melons for statistical analysis, in order to obtain a simplified view of the total aroma characteristics of the melon samples. The first three principal components accounted for 76.84% of the total explained variance between the samples. Rotated principal component loadings for aroma terms are shown in Table 3. Loading values (i.e., correlation coefficients) >0.800 are marked throughout in bold type. The volatile compounds that characterized the aroma profile of La Mancha Trujillo melons, based on mean scores and standard deviations for the means concentrations of volatile compounds displaying the strongest correlation (>0.8) with components 1, 2 and 3 for each of the three years, are shown in Figure 1, which illustrates the simultaneous projection of the 6 melons and the 30 volatile aroma compounds. According to these loading values, eugenol, 4-methylguaiacol, decanoic acid, benzaldehyde, 4-vinylguaiacol, 3-octanone, (*E,Z*)-2,6-nonadienal, 1-octanol, isoeugenol, 1,2-benzothiazole, 1-nonanol, (*Z*)-3-nonen-1-ol, (Z)-6-nonen-1-ol, (E)-6-nonen-1-ol, nonanal, (Z)-6-nonenal, 2-ethyl-1-hexanol, geraniol, nerol, 3-hydroxyethyl butyrate, (*Z,Z*)-3,6-nonadien-1-ol, (*E*)-2-hexenal, benzyl alcohol, vanillin, benzoic acid, (*Z*)-2-nonenal, hexanal, hexenoic acid, 1-hexanol and isoeugenol were considered the major volatile compounds of aroma of La Mancha Trujillo melons.

### 3.3. Quantitative Descriptive Sensory Analysis

#### 3.3.1. Olfactory Profile

Table 4 shows mean aroma-intensity scores and standard deviations. As well as within compound concentrations. it was applied a Student–Newman–Keuls test to discriminate between means for sensory data. The results suggested that aroma of La Mancha Trujillo melons was characterized by higher intensities of sweet and fresh fruit aromas whit honey. cucumber. jam/marmalade. green and ripe fruit notes. However. certain differences were perceived by the panel in the aroma profiles of Trujillo melons of different harvests. The aroma sensory profile of 2012 La Mancha Trujillo melons has slightly more pronounced aroma attributes.

#### 3.3.2. Gustatory Profile

Table 5 shows mean aroma-intensity scores and standard deviations. In order to discriminate between means for sensory data. the Student–Newman–Keuls test was applied. Flavors by mouth of sweet. green. cucumber. ripe fruit. spicy. honey. fresh fruit and jam/marmalade were the ones which presented a higher intensity in La Mancha Trujillo melons. All harvests shared similar flavor features. and it can be highlighted that honey and cucumber aromas were more pronounced than sweet. spicy and green aromas in all harvests studied.

### 3.4. PLS Modeling Relationship between Sensory Descriptors (Aroma) and Volatile Compounds of Melons

With the aim of establishing correlations between the aroma sensory attributes of La Mancha Trujillo melons and the volatile compounds that characterized the aroma profile of La Mancha Trujillo melons that displayed the strongest correlation (>0.8). it was a partial least squares regression (PLS) was established with components 1, 2 and 3 for each of the three years (see Table 3).

**Figure 2:**
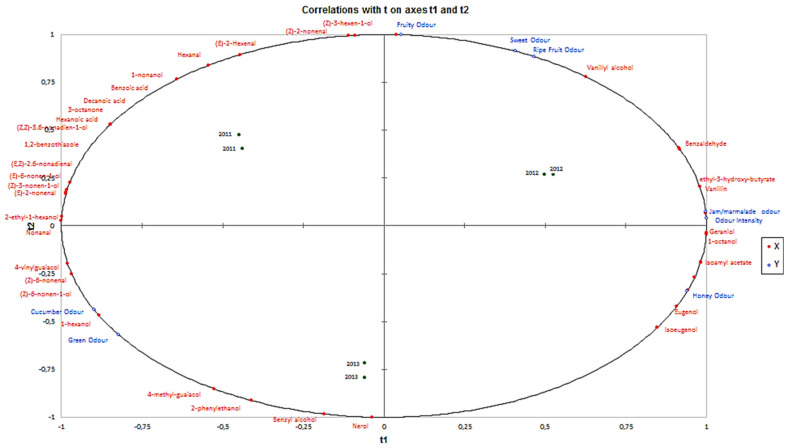
PLS loading for t1 y t2 for sensory attributes (Y variables) and volatile compounds of La Mancha Trujillo melons (X variables) from three harvest considered.

Figure 2 shows PLS made considering aroma sensory attributes and volatiles compounds that characterized the volatile profile of La Mancha Trujillo melons. Based on proximity on the left side of the plot, it can be observed that the aroma sensory attributes cucumber and green odour were positively related to the presence of 1-hexanol, (*Z*)-6-nonenal, (*Z*)-6-nonen-1-ol, nonanal, 4-vinylguaiacol, 2-ethyl-1-hexanol, (*E*)-2-nonenal, (*Z*)-3-nonen-1-ol, (*E*)-6-nonen-1-ol, *(E.Z)*-2.6-nonadienal, (*E*)-2-hexenal, (*Z*)-2-nonenal, hexanal, 1.2-benzothiazole, (*Z.Z*)-3.6-nonadien-1-ol, hexanoic acid, 3-octanone, decanoic acid, benzoic acid. 1-nonanol, nerol, benzyl alcohol and 2-phenylethanol with a high correlation coefficient (mainly between 0.60 and 0.85). In contrast. sweet, fruity, ripe fruit, jam/marmalade, odour intensity and honey attributes are located on the right side of the plot and were mainly correlated with eugenol, isoeugenol, isoamyl acetate, geraniol, 1-octanol, vanillinic alcohol, benzaldehyde and 3-hydroxyethyl butyrate.

PLS component 1 contributed to the spread of samples on the left side of the plot, which was mainly based on the intensity of cucumber and green odours, in opposition to samples on the right side, which were more sweet, fruity, ripe fruit, jam/marmalade, odour intensity and honey. Melons from 2011 and 2013 harvest are located on the left side of the plot and showed higher intensity cucumber and green aroma sensory attributes. These attributes are positively correlated with C_6_ compounds and aldehydes with low molecular weight. On the other hand, melons from the 2012 harvest are located on the right side of the plot and are characterized by their aroma intensity and their high intensity of sweet, fruity, ripe fruit, jam/marmalade and honey aroma sensory attributes. These aroma sensory attributes are influenced by the concentration of certain terpenes such as geraniol, benzene compounds such benzaldehyde, vanillin, vanillic alcohol, eugenol and isoeugenol and the esters isoamyl acetate and 3-hydroxyethyl butyrate, which are principally related with sweet and fruity aromas sensory attributes.

## 4. Conclusions

With this work, a better knowledge of the aroma composition and sensory profile of Trujillo melons grown in the La Mancha region of Spain has been achieved. In addition. this study presents the results obtained from the first experiment performed on the free aroma compounds from this melon type from the Castilla-La Mancha region. Three conclusions can reached: firstly, that the free aroma of La Mancha Trujillo melons is characterized by a high concentration of aldehydes, alcohols, acids and benzene compounds. The sensory aroma profile of Trujillo melons was characterized by jam/marmalade, green, cucumber, fresh fruit, honey, sweet and ripe fruit aroma descriptors. Lastly, the gustatory profile of Trujillo melons was characterized by sweet, honey, jam/marmalade, cucumber, fresh fruit ripe fruit and green flavor-by-mouth descriptors.

This study showed that this melon variety presents a good aroma that favors the differentiation of La Mancha melons on the national and international market and can diversify the offer to the consumer.

## Figures and Tables

**Table 1:** General composition of La Mancha Trujillo melons. Mean values and relative standard deviations (*n* = 2).

		Harvest	
	2011	2012	2013
pH	4.23 ^a^ (0.03)	5.63 ^b^ (0.24)	5.80 ^b^ (0.44)
Titrable acidity (mg citric acid/L)	1280 ^a^ (1.72)	860 ^b^ (0.81)	825 ^b^ (0.91)
Total soluble solids (°Brix)	11.4 ^a^ (0.18)	15.0 ^b^ (0.45)	15.7 ^b^ (0.24)

^a, b^ Different superscripts in the same row indicate statistical differences at the 0.05 level according to the Student-Newman-Keuls test.

**Table 2:** Volatile compounds of La Mancha Trujillo melons. Mean concentration (µg/L) and relative standard deviations (*n* = 2).

			Harvest		
RI	Source	Compounds	2011	2012	2013
		*Aldehydes*			
1086	Sigma-Aldrich	Hexanal	2.44 ^c^ (0.99)	1.85 ^b^ (5.58)	1.21 ^a^ (5.42)
1227	Sigma-Aldrich	(*E*)-2-hexenal	2.54 ^a^ (1.57)	2.43 ^a^ (1.31)	1.84 ^b^ (11.7)
1150	Sigma-Aldrich	(*E*)-2-heptenal	2.25 ^a^ (0.60)	2.37 ^a^ (4.12)	2.44 ^a^ (7.67)
1390	Sigma-Aldrich	Nonanal	7.49 ^a^ (1.99)	8.63 ^a^ (11.7)	8.37 ^a^ (7.77)
1552	Sigma-Aldrich	(*Z*)-6-nonenal	2.58 ^a^ (0.51)	2.33 ^a^ (4.78)	2.52 ^a^ (6.20)
1015	Tentatively Identified	(*E,E*)-2,4-heptanedial	0.63 ^a^ (0.37)	0.70 ^a^ (0.41)	0.54 ^a^ (0.38)
1345	Tentatively Identified	(*Z*)-2-nonenal	2.37 ^b^ (1.37)	2.24 ^b^ (0.39)	1.43 ^a^ (0.41)
1537	Sigma-Aldrich	(*E*)-2-nonenal	2.36 ^a^ (1.67)	3.11 ^b^ (2.21)	2.89 ^b^ (1.42)
1588	Sigma-Aldrich	(*E,Z*)-2,6-nonadienal	114.31 ^a^ (0.72)	108.23 ^a^ (7.52)	110.43 ^a^ (4.95)
		*Alcohols*			
1168	Sigma-Aldrich	3-penten-1-ol	18.51 ^a^ (10.72)	20.42 ^a^ (3.42)	19.71 ^a^ (1.00)
1180	Sigma-Aldrich	2-methyl-1-butanol	1.65 ^a^ (1.35)	1.31 ^a^ (0.54)	1.20 ^a^ (0.32)
1328	Sigma-Aldrich	4-methyl-1-pentanol	10.56 ^a^ (3.38)	12.12 ^a^ (1.39)	11.13 ^a^(1. 12)
1341	Sigma-Aldrich	3-methyl-1-pentanol	2.61 ^a^ (1.02)	3.74 ^a^ (2.46)	3.11 ^a^ (2.32)
1282	Merck	1-hexanol	3.85 ^a^ (2.21)	2.42 ^b^ (1.37)	3.15 ^c^ (0.04)
1296	Sigma-Aldrich	(*Z*)-3-hexen-1-ol	3.40 ^a^ (1.86)	3.40 ^a^ (2.86)	3.08 ^a^ (1.81)
1348	Sigma-Aldrich	1-heptanol	5.81 ^a^ (1.16)	5.74 ^a^ (2.75)	5.62 ^a^ (1.03)
1452	Sigma-Aldrich	1-octanol	1.05 ^a^ (0.82)	1.11 ^a^ (0.82)	1.08 ^a^ (0.94)
1405	Merck	2-ethyl-1-hexanol	10.17 ^a^ (8.12)	9.54 ^a^ (0.70)	9.85 ^a^ (3.85)
1554	Merck	1-nonanol	16.22 ^a^ (2.12)	14.93 ^b^ (0.49)	14.35 ^b^ (1.13)
1680	Sigma Aldrich	(*Z*)-3-nonen-1-ol	97.51 ^a^ (3.38)	104.65 ^b^ (5.42)	104.54 ^b^ (5.42)
1710	Sigma Aldrich	(*Z*)-6-nonen-1-ol	6.45 ^a^ (7.73)	5.42 ^b^ (2.97)	5.82 ^b^ (1.77)
1782	Sigma Aldrich	(*E*)-6-nonen-1-ol	18.80 ^a^ (7.24)	16.91 ^b^ (7.75)	17.62 ^b^ (2.40)
1742	Tentatively Identified	(*Z,Z*)-3,6-nonadien-1-ol	235.78 ^a^ (10.3)	210.56 ^b^ (8.47)	210.63 ^b^ (5.93)
		*Acids*			
1426	Sigma Aldrich	Acetic acid	0.82 ^a^ (0.02)	0.97 ^b^ (0.23)	0.84 ^a^ (0.05)
1546	Sigma Aldrich	Propanoic acid	1.74 ^a^ (0.32)	1.68 ^a^ (0.28)	1.62 ^a^ (0.14)
1583	Sigma Aldrich	Isobutanoic acid	3.25 ^a^ (1.10)	2.56 ^a^ (2.17)	2.56 ^a^ (1.17)
1816	Sigma Aldrich	Hexanoic acid	8.19 ^a^ (1.14)	7.88 ^a^ (1.24)	7.23 ^a^ (3.22)
1929	Sigma Aldrich	Heptanoic acid	3.68 ^a^ (0.95)	2.52 ^b^ (0.51)	3.43 ^a^ (0.40)
2024	Sigma Aldrich	Octanoic acid	13.32 ^a^ (4.38)	12.75 ^b^ (1.44)	12.32 ^c^ (0.44)
2202	Sigma Aldrich	Nonanoic acid	37.12 ^a^ (11.02)	33.81 ^b^ (8.94)	27.6 ^c^ (3.21)
2289	Sigma Aldrich	Decanoic acid	8.02 ^a^ (0.14)	7.83 ^a^ (1.56)	7.41 ^a^ (0.42)
2439	Sigma Aldrich	Dodecanoic acid	13.81 ^a^ (1.36)	13.12 ^a^ (2.46)	13.18 ^a^ (1.21)
2724	Sigma Aldrich	Tetradecanoic acid	47.32 ^a^ (4.18)	42.31 ^b^ (12.2)	44.27 ^c^ (5.63)
2786	Sigma Aldrich	Pentadecanoic acid	29.91 ^a^ (8.54)	26.75 ^a^ (7.17)	26.17 ^a^ (0.87)
		*Benzenic compounds*			
1530	Sigma Aldrich	Benzaldehyde	5.18 ^a^ (8.82)	5.30 ^a^ (5.74)	5.19 ^a^ (7.22)
1871	Merck	Benzyl alcohol	26.31 ^a^ (8.93)	25.02 ^a^ (2.25)	33.21 ^b^ (6.16)
1892	Sigma Aldrich	2-phenylethanol	8.28 ^a^ (3.79)	8.09 ^a^ (1.19)	8.59 ^a^ (1.54)
1899	Tentatively Identified	1,2-benzothiazole	1.27 ^a^ (0.98)	1.25 ^a^ (0.23)	1.15 ^a^ (1.04)
1933	Tentatively Identified	Phenol	8.25 ^a^ (4.91)	7.94 ^a^ (0.52)	8.06 ^a^ (1.70)
1971	Sigma Aldrich	4-methyl-guaiacol	8.00 ^a^ (0.26)	7.21 ^a,b^ (1.62)	8.81 ^b^ (5.64)
2193	Sigma Aldrich	Eugenol	9.07 ^a^ (1.82)	9.51 ^a^ (8.30)	9.45 ^a^ (7.41)
2219	Sigma Aldrich	4-vinylguaiacol	0.72 ^a^ (4.41)	0.55 ^a^ (9.02)	0.67 ^a^ (5.24)
2378	Sigma Aldrich	Benzoic acid	2.62 ^a^ (0.82)	2.13 ^a^ (0.44)	1.77 ^b^ (0.78)
2302	Sigma Aldrich	Isoeugenol	6.27 ^a^ (1.28)	6.90 ^a^ (1.41)	6.59 ^a^ (1.30)
2511	Sigma Aldrich	Vanillin	14.12 ^a^ (1.82)	24.12 ^b^ (3.69)	18.25 ^c^ (11.52)
2936	Tentatively Identified	Vanillyl alcohol	11.42 ^a^ (1.05)	11.27 ^a^ (1.40)	11.22 ^a^ (2.56)
		*Terpenic compounds*			
1755	Sigma Aldrich	Nerol	1.29 ^a^ (0.55)	1.22 ^a^ (1.32)	1.31 ^a^ (1.04)
1831	Sigma Aldrich	Geraniol	0.59 ^a^ (0.09)	0.61 ^a^ (0.18)	0.60 ^a^ (0.29)
		*Esters*			
1145	Merck	Isoamyl acetate	1.86 ^a^ (0.06)	2.96 ^b^ (0.78)	2.56 ^b^ (1.06)
1432	Merck	Ethyl octanoate	Tr	Tr	Tr
1499	Tentatively Identified	3-hydroxy ethyl-butyrate	0.54 ^a^ (0.28)	0.61 ^a^ (0.19)	0.56 ^a^ (0.13)
		*Ketones*			
1114	Tentatively Identified	(*E*)-3-penten-2-one	7.70 ^a^ (3.88)	7.43 ^a^ (2.13)	8.41 ^a^ (1.51)
1277	Tentatively Identified	3-octanone	0.30 ^a^ (0.68)	0.28 ^a^ (0.77)	0.21 ^a^ (0.13)
15030	Tentatively Identified	1-(1-cyclohexen-1-yl)-ethanone	1.44 ^a^ (1.00)	1.28 ^a^ (0.20)	1.22 ^a^ (1.10)
2011	Tentatively Identified	5-pentyl-dihydro-2(3H)-furanone	5.38 ^a^ (1.11)	5.25 ^a^ (0.80)	5.50 ^a^ (1.26)

Tr: Traces.^a, b, c,^ Different superscripts in the same row indicate statistical differences at the 0.05 level according to the Student-Newman-Keuls test.

**Table 3:** Rotated principal component loadings for volatile compounds of La Mancha Trujillo melons.

Volatile Compounds.	Components		
	1	2	3
Eugenol	**−0.98**	−0.02	−0.109
4-Methylguaiacol	**−0.97**	−0.09	−0.138
Decanoic acid	**0.97**	0.09	0.139
4-Vinylguaiacol	**−0.96**	0.2	−0.098
3-Octanone	**0.96**	0.17	0.146
(*E,Z*)-2.6-Nonadienal	**0.93**	0.31	0.1
1-Octanol	**−0.92**	−0.23	−0.056
Isoeugenol	**−0.92**	−0.24	−0.056
(Z)-3-hexen-1-ol	**0.91**	−0.37	0.15
1.2-Benzothiazole	**0.86**	−0.41	0.057
1-Nonanol	**0.85**	0.17	0.211
(*Z*)-3-Nonen-1-ol	**−0.85**	−0.40	−0.164
(*Z*)-3-Hexen-1-ol	**−0.84**	−0.36	−0.163
(*Z*)-6-Nonen-1-ol	**0.80**	0.57	0.166
Nonanal	**−0.80**	−0.55	−0.123
Vanillyl alcohol	**−0.76**	0.25	0.031
1-Hexanol	−0.78	0.45	0.051
(*Z*)-6-Nonenal	0.03	**0.98**	−0.043
2-Ethyl-1-hexanol	−0.07	**0.97**	0.011
Geraniol	0.12	**−0.97**	−0.009
Nerol	0.13	**−0.98**	0.032
Phenol	0.01	**0.96**	0.039
Benzaldehyde	−0.06	**−0.94**	0.076
Ethyl 3-hydroxy-butyrate	−0.21	**−0.95**	0.048
Vanillin	−0.21	**−0.80**	−0.134
(*E*)-6-Nonen-1-ol	0.60	**0.76**	0.091
Hexanoic acid	−0.48	**0.73**	0.034
(*Z,Z*)-3.6-Nonadien-1-ol	0.56	0.77	0.162
(*E*)-2-Hexenal	0.18	0.13	**0.947**
Benzyl alcohol	0.00	0.20	**−0.922**
Benzoic acid	0.29	0.06	**0.922**
(*Z*)-2-Nonenal	0.13	0.14	**0.885**
Hexanal	0.19	0.03	**0.841**

Loading values (i.e., correlation coefficients) >0.800 are marked throughout in bold type.

**Table 4:** Olfactive profile of La Mancha Trujillo melons scores of 15 judges and standard deviations (two replicates).

Odour Descriptors	Vintage		
	2011	2012	2013
Odour intensity	6.03 ^a^ (0.69)	7.58 ^b^ (0.41)	6.70 ^a^ (1.08)
Sweet	5.33 ^a^ (0.69)	6.69 ^b^ (0.88)	4.88 ^a^ (0.55)
Cucumber	2.26 ^a^ (0.51)	0.00 ^b^ (0.00)	2.18 ^a^ (0.61)
Ripe fruit	1.35 ^a^ (0.13)	2.38 ^b^ (0.85)	0.00 ^c^ (0.00)
Green	3.05 ^a^ (0.88)	1.86 ^b^ (0.85)	3.23 ^a^ (0.10)
Fresh fruit	5.20 ^a^ (0.69)	5.23 ^a^ (0.17)	4.99 ^a^ (0.53)
Honey	0.00 ^b^ (0.00)	2.36 ^a^ (0.73)	1.84 ^a^ (0.78)
Jam/marmalade	0.00 ^a^ (0.00)	2.66 ^b^ (0.65)	1.08 ^c^ (0.55)

^a. b. c^ Different superscripts in the same row indicate statistical differences at the 0.05 level according to the Student-Newman-Keuls test.

**Table 5:** Mean gustative profile of melon scores of 15 judges and standard deviations (two replicates).

Flavour-by-Mouth	Vintage		
	2011	2012	2013
Taste intensity	6.43 ^a^ (0.86)	7.66 ^b^ (1.02)	6.73 ^a^ (1.01)
Sweet	6.14 ^a^ (0.83)	6.84 ^a^ (0.93)	4.81 ^b^ (0.36)
Green	1.76 ^a^ (0.87)	0.61 ^b^ (0.92)	2.41 ^a^ (1.02)
Cucumber	1.16 ^a^ (0.97)	0.00 ^a^ (0.00)	0.93 ^a^ (0.62)
Ripe Fruit	1.73 ^a^ (0.68)	1.03 ^a^ (0.26)	0.00 ^b^ (0.00)
Spicy	0.94 ^a^ (0.33)	0.41 ^a^ (0.58)	0.00 ^a^ (0.00)
Honey	2.81 ^a^ (0.69)	3.18 ^a^ (0.30)	2.08 ^a^ (0.43)
Jam/marmalade	0.00 ^a^ (0.00)	3.12 ^b^ (1.00)	1.34 ^c^ (0.94)
Fresh fruit	4.76 ^a^ (0.14)	4.56 ^a^ (0.20)	4.39 ^a^ (0.65)
Acidity	5.00 ^a^ (0.00)	4.33 ^b^ (0.35)	5.00 ^a^ (0.00)
After-Taste intensity	6.64 ^a^ (0.46)	7.30 ^a^ (0.13)	6.33 ^a^ (0.74)
After-Taste quality	6.82 ^a^ (0.56)	7.54 ^a^ (0.76)	6.50 ^a^ (0.33)

^a. b. c^ Different superscripts in the same row indicate statistical differences at the 0.05 level according to the Student-Newman-Keuls test.
